# 3D surface texture analysis of high‐resolution normal fields for facial skin condition assessment

**DOI:** 10.1111/srt.12793

**Published:** 2019-09-28

**Authors:** Alassane Seck, Hannah Dee, William Smith, Bernard Tiddeman

**Affiliations:** ^1^ Aberystwyth University Aberystwyth UK; ^2^ University of York York UK; ^3^ Present address: ARM Ltd Cambridge UK

**Keywords:** 3D surface texture, 3D capture, skin analysis, texture

## Abstract

**Background:**

This paper investigates the use of a *light stage* to capture high‐resolution, 3D facial surface textures and proposes novel methods to use the data for skin condition assessment.

**Materials and Methods:**

We introduce new methods for analysing 3D surface texture using high‐resolution normal fields and apply these to the detection and assessment of skin conditions in human faces, specifically wrinkles, pores and acne. The use of high‐resolution normal maps as input to our texture measures enables us to investigate the 3D nature of texture, while retaining aspects of some well‐known 2D texture measures. The main contributions are as follows: the introduction of three novel methods for extracting texture descriptors from high‐resolution surface orientation fields; a comparative study of 2D and 3D skin texture analysis techniques; and an extensive data set of high‐resolution 3D facial scans presenting various skin conditions, with human ratings as “ground truth.”

**Results:**

Our results demonstrate an improvement on state‐of‐the‐art methods for the analysis of pores and comparable results to the state of the art for wrinkles and acne using a considerably more compact model.

**Conclusions:**

The use of high‐resolution normal maps, captured by a light stage, and the methods described, represent an important new set of tools in the analysis of skin texture.

## 1 INTRODUCTION

Computer‐aided skin condition assessment has been mostly addressed using two‐dimensional texture analysis techniques on skin images or coarse geometrical features extracted from the skin's three‐dimensional macro‐structures. The first trend ignores the three‐dimensional nature characterising most skin conditions, and the latter mainly deals with geometrical features that are not fine enough to capture skin structures at the meso‐ and micro‐scales. However, advances in three‐dimensional surface imaging have recently opened up the possibility of capturing the fine geometrical structures of human skin, along with its reflectance properties. These can now be recovered with unprecedented quality and resolution (down to the level of individual pores).

The methods proposed in this work aim at exploiting these advances and revisiting the formulation of texture analysis as a three‐dimensional problem. For data collection, we have used a *light stage* to capture high‐resolution facial normal fields along with their reflectance properties. The collected data are photo‐realistically rendered and presented to the general public for annotations indicating the presence of the studied skin conditions. These constitute the ground truth upon which the proposed methods are applied in order to learn models for detecting and assessing facial skin conditions. We compare our three methods on this new data set, including BTF Texton results as a gold‐standard method, and classical 2D‐texture measures (with 3D enhancements) as a baseline method.

## 2 LITERATURE REVIEW

### 2.1 2D texture analysis

Texture characterisation is key to a number of visual computing‐related applications such as object recognition, content‐based image retrieval and computer graphics. A number of efficient and powerful 2D texture analysis methods have been proposed in the literature. These methods can be divided into three categories:

- *Statistical methods* which assume that the texture is fully determined by the spatial distribution of pixel values in the image. Examples of statistical methods include the use of the Grey Level Co‐occurrence Matrix,^1^ the Autocorrelation function, the Symmetric Auto‐correlation function (SAC) and its extensions (SRAC and SCOV)^2^ and the well‐known Local Binary Patterns (LBPs).^3^, ^4^, ^5^
- *Structural methods* that consider texture as a structured layout of texture primitives also called texture elements. Such methods divide into geometrical and topological approaches. In geometrical approaches, coarse geometrical properties such as perimeter and compactness are used to characterise texture primitives.^6^ Topological approaches use various filtering methods to extract primitives such as lines, edges and blobs. The texture descriptor is then made of different properties of these extracted primitives, namely number, orientation and density.^7^, ^8^
- *Model‐based methods* in which the texture is represented with either a probabilistic model or a projective decomposition along a set of basis functions. These representations require the determination of a certain number of parameters or coefficients to characterise the texture. The Markov model‐based methods constitute an important subset of these methods. Hidden Markov Models (HMMs) have been extensively used to characterise texture.^9^, ^10^ Cohen et al used a Gaussian Markov Random Field (GMRF) to model rotated and scaled texture.^11^ Methods using sub‐band decomposition techniques include the wavelet transform,^12^, ^13^ the steerable pyramid^14^ and the Gabor Bank of filters.^13^, ^15^, ^16^

The approach chosen generally depends upon the aspect of texture one wishes to capture. All 2D methods make the implicit assumption that apparent texture is independent of illumination and viewpoint. While this assumption can be approximated when studying smooth surfaces, the apparent texture of surfaces involving rough relief is more obviously illumination‐ and viewpoint‐dependent.

### 2.2 3D surface texture analysis

The appearance of a natural surface is not only determined by intrinsic reflectance properties (colour or albedo), but is also considerably affected by the interaction between geometrical structure, light and viewpoint. Various methods have been proposed to capture aspects of this variability. In the rest of this paper, we will refer to these types of texture methods, responsive to illumination/view changes, as 3D Surface Texture. These can be categorised into three families: 3D Texton‐based methods, Bidirectional Texture‐based methods and Geometrical methods.

- *3D Texton‐based methods*: The notion of a 3D Texton was introduced by Leung and Malik 17 and has been widely used and extended to represent natural surfaces’ visual appearance. The main idea is to simultaneously encode the two attributes that most affect how a surface is visually perceived; these are the surface normals and reflectance properties. To characterise a given surface's texture, the approach exploits filter responses on several images of the same surface taken in different imaging conditions (illumination and viewpoint). In addition, these filter responses are quantised into a reduced set of texture prototypes. This results in a dictionary of tiny texture patch representations called 3D textons that cover all possible local surface configurations.
- *Bidirectional Texture‐based methods*: In contrast to the 3D texton‐based methods, the Bidirectional Texture Function (BTF) operates at a higher level of abstraction representing surface properties that affect the apparent texture. This makes them useful for analysing as well as for synthesising natural texture (when used for analysis, they are generally combined with a texton‐based quantisation layer). The notion of a BTF was first introduced by Dana et al 18 and has been called the most advanced and accurate representation of natural surfaces visual properties to date.^19^ The BTF models a surface's texture as a function of illumination and viewpoint. It is a seven‐dimensional function and represents texture as a function of the spectral band, the planar position, the view and light directions:

(1) $$BTF\left(r_{x},r_{y},\rho ,\phi_{i},\theta_{i},\phi_{v},\theta_{v}\right)$$

where $r_{x}$ and $r_{y}$ are the horizontal and vertical positions, respectively, ρ is the spectral band, $\phi_{i}$ and $\theta_{i}$ are the elevation and azimuthal angles of the light direction, respectively, and $\phi_{v}$ and $\theta_{v}$ the elevation and azimuthal angles of the viewing direction, respectively.
BTF measurement generally involves a complex capture set‐up in which automated devices coordinate changes in either the lighting conditions or the camera viewpoint or, in some systems, both.^18^, ^20^, ^21^, ^22^ Although BTF is extensively used in Computer Graphics, generally for photo‐realistic texture synthesis and rendering purposes, it is also used to create and evaluate texture features that are robust to imaging conditions. Dana et al analysed skin texture using a BTF made of more than 3500 images to discriminate between skin disorders such as acne and psoriasis.^23^
Suen and Healey introduced the notion of dimensionality surface as a measure of appearance variability due to the effects of viewpoint and illumination changes on fine surface geometry.^24^ From the CURet Bidirectional Texture database,^18^ they applied a set of multi‐band correlation functions $R_{\mathrm{ij}}\left(m,n\right)$ on each image of each material sample (i and j being spectral bands and $\left[m;n\right]$ an image region).
Caputo et al introduced the KTH‐TIPS2 material database (11 materials each with four different imaging conditions) and used it to test the robustness of various state‐of‐the‐art texture descriptors to pose and illumination change.^25^ They experimented with including various numbers of pose and illumination conditions in their training set, and testing with samples from unseen pose/illumination conditions. One of their findings was that the more sample groups they add to the training set the better the classification method performs. More recent studies include the work of Liu et al in which they propose learning discriminative models for determining optimal texture filters for given illumination conditions.^26^ The authors collected a BTF database using a dome of controllable LEDs and a fixed camera. The acquired database consists of 90 material samples captured under 6 spectral bands and 25 lighting directions.
*Geometric methods: *The methods presented in the two preceding sections are image‐based as the intrinsic geometry of the material's surface is not known. The considerable number of image samples needed by these methods in order to capture the three‐dimensional properties of the studied surfaces makes their use demanding in storage capacity. Some recent works have looked at characterising 3D texture directly from measured fine geometry, providing a more compact representation of the intrinsic three‐dimensional properties. Smith et al propose computing a co‐occurrence matrix from the orientation of measured surface normals.^27^ Their method involves quantising the normals’ orientation into a discrete space. For each normal, the slant and tilt angles are discretised in three equal intervals. This result in 9 levels upon which the co‐occurrence matrix is constructed. Sandbach et al extracted Local Binary Pattern features from two different 2D representations of 3D geometrical data to classify 3D facial action units.^28^ The two representations are a simple depth map and the Azimuthal Projection Distance Image. This latter representation encodes the 3D surface orientation in a 2D greyscale image, by projecting each surface normal onto the tangent plane and taking the L norm of the projected point as a grey level.

### 2.3 3D skin micro‐structure imaging

There are a family of techniques which concentrate not on general 3D surface texture, but on the specific problem of human skin micro‐structure, motivated by medical (dermatological) applications and the increasing demand for photo‐realistic solutions from the game and film industry. Cula et al used a bidirectional imaging system to capture the micro‐structure of skin regions affected by diverse dermatological disorders (psoriasis, acne, contact dermatitis etc)^23^ and released these 3500 images as the Rutgers Skin Texture Database. They used two different mechanical set‐ups that allowed them to capture skin regions in various viewpoints and light directions. Hong and Lee^29^ used a mobile phone and a mirror system to capture and analyse acne in 3D. Zhou et al^30^ captured 3D data of skin surfaces using a photometric stereo device and analysed them using differential geometry features and a linear classifier to classify malignant melanomas and benign lesions.

Ma et al use a *light stage* to capture three‐dimensional facial skin structure down to the level of the pores.^31^ They combined this with a polarised light technique to separate the diffuse and specular surface properties. The resulting data are in the form of normal maps. They have shown that specular normal maps capture most of the surface detail while the diffuse maps are more subject to subsurface scattering. These polarisation and wavelength‐dependent measurements constitute very useful data for understanding how the human skin interacts with light as well modelling its micro‐structure.

Many improvements and applications have been added to the capture system since. Graham et al proposed a measurement‐based synthesis of facial microgeometry.^32^ The authors measure the micro‐structure of skin patches using a twelve‐light hemisphere able to emit cross‐polarised light. The acquired skin micro‐structure images are processed to extract displacement maps. Another skin reflectance measurement using a *light stage* is conducted by Weyrich et al.^33^ They augment their data with an extra skin subsurface scan using a fibre optic spectrometer which is a device allowing measurements of subsurface properties such as haemoglobin or glucose concentrations. The authors also fitted the analytic BSSRDF (Bidirectional Subsurface Reflectance Distribution Function) proposed by Jensen et al^34^ to their measured data and conducted analysis on the relations between the BSSRDF parameters (scattering and absorption coefficients) and various attributes of the subject such as age and skin type.

PRIMO (http://www.gfm3d.com/) is a commercial solution for 3D skin measurements used in some automated skin disruption detection studies such as Choi et al.^35^ It is a hand‐held optical‐based system using structured light and a high‐resolution sensor allowing measurements of skin micro‐topography and roughness with a field of view of 45×30×30mm. The Anterra 3D (http://miravex.com/antera-3d/) is another hand‐held commercial system for 3D skin imaging and measurement. Messaraa et al^36^ compared skin health measurements such as roughness and wrinkle length/depth from Anterra 3D with a 2D imaging and image analysis (using DermaTOP and image analysis on parallel‐polarised images). The results showed good correlation between the 3D and 2D measurements, and the ability to detect changes due to application of a cosmetic product.

### 2.4 Literature review summary

In the previous sections, the state of the art in 2D/3D texture analysis and human skin micro‐structure imaging techniques were introduced. It is clear that advances have been made in face imaging technology as it is now possible to capture the skin's three‐dimensional micro‐structure down to the level of pores. However, it seems that these newly available possibilities for data capture are not fully exploited on the analysis side, as most of the studies presented above use either two‐dimensional image‐based texture features or rather coarse three‐dimensional surface properties. One of the few studies that exploited the skin three‐dimensional micro‐structure used a BTF representation 18 which takes into account changes in illumination and viewpoint, but is still an image‐based representation as the underlying surface geometry is not known.

## 3 3D MEASURES FOR SKIN TEXTURE CHARACTERISATION

In this paper, we introduce here three novel 3D surface texture analysis methods: the rotation fields pyramid; Local Orientation Patterns; and Multi‐scale Azimuthal Projection distance. These take full advantage of the recent advances made in photometric stereo imaging techniques. In contrast to image‐based methods, these operate directly on the skin geometrical fine structure captured in the form of surface normal fields captured using a *light stage*. We compare our novel 3D methods with both classic 2D texture descriptors and simplistic 3D extensions of these.

### 3.1 Extensions of existing 2D descriptors to 3D

Before introducing our three proposed 3D texture descriptors, we describe here how a number of standard 2D feature extraction methods can be extended to 3D analysis, in order to provide a set of comparable baseline methods. We experiment with two widely used 2D texture descriptors, namely the Gabor filter bank 16, 37 and rotation invariant LBPs.^2^ Although the normal map estimated by the light stage can be represented in a 3‐channel image, with the RGB channels being used to store the normal's x, y and z components, operating on them with filters etc does not correctly account for the non‐linear manifold on which the normals lie. Instead of calculating the texture measures introduced above directly on the normal maps, we propose deriving these from either the slant‐tilt space or the tangent space.

#### 3.1.1 Slant‐tilt space

**Figure 1:**
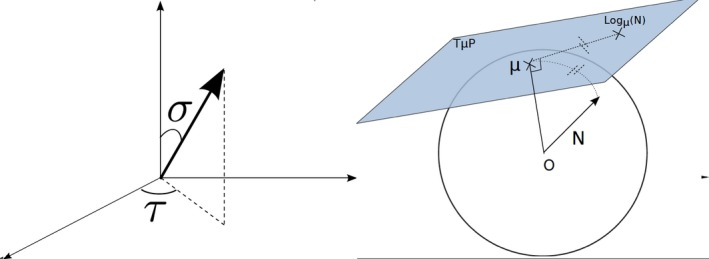
On the left, the normal's slant σ and tilt τ. On the right, projection of a normal onto the local tangent plane [Colour figure can be viewed at http://www.wileyonlinelibrary.com]

The normal's slant and tilt are extracted at each position (Figure 1). This results in a map which contains two values corresponding to the normal's elevation and azimuth at each position. We keep the tangent values so the slant‐tilt map is normalised in $\left[-1,1\right]$. Considering $n=\left(n_{x},n_{y},n_{z}\right)$ denoting a normal, the slant and tilt tangent values are obtained with:

(2) $$\tan\sigma =\frac{n_{x}^{2}+n_{y}^{2}}{n_{z}},\tan\tau =\frac{n_{y}}{n_{x}}$$

#### 3.1.2 Tangent space

In this approach, the normals are considered as elements of a Riemannian manifold and these are unfolded about the local means using a logarithmic mapping (Figure 1). This results in a tangent map whose elements are 2‐dimensional coordinates and are obtained with:

(3) $$\log_{\mu_{\theta_{0},\tau_{0}}}\left(n_{\theta ,\tau }\right):\left\{\begin{array}{cc} x^{\prime }=k & \cos\theta \sin\left(\tau -\tau_{0}\right)\\ y^{\prime }=k & \cos\theta_{0}\sin\tau -\sin\theta_{0}\cos\theta \cos\left(\tau -\tau_{0}\right)) \end{array}\right.$$

where $\theta =\frac{\pi }{2}-\sigma$. $\theta_{0}$ and $\tau_{0}$ are the spherical coordinates of the local normal mean μ. At each neighbourhood, the local normal mean is the one that minimises the mean of the geodesic distances to all the other normals in the same neighbourhood.

### 3.2 3D surface texture characterisation

We adopt a multi‐scale scheme where at each level, the texture filter (either Gabor filters 16, 37 or rotation invariant LBPs^2^) is applied on either the slant‐tilt map or the tangent map. This results in two responses, one for each channel. The responses are normalised to the interval $\left[0,1\right]$. Assuming $R^{c,l}$ denotes the response on the channel c at the level l, the normalisation is performed with:

(4) $$R_{\text{normalized}}^{c,l}=\frac{R^{c,l}-\min R^{c,l}}{\max R^{c,l}-\min R^{c,l}}$$

The histograms of the two normalised responses are computed and concatenated to form the texture descriptor at level l. The same process is repeated at the subsequent level with a down‐sampled version of the current normal map. As previously mentioned, a convolution should not be done directly on the normals (because they do not occupy a linear space), so the down‐sampling is done in the tangent plane with a Gaussian low pass, followed by projecting the result back into the original 3‐dimensional space using the manifold exponential chart.

### 3.3 Feature extraction and classification

For each sample, we build a 3‐level multi‐scale feature pyramid. The Gabor filter bank and R‐LBPs are applied on the albedo samples, and their extensions to 3D are used on the corresponding normal map samples in the slant/tilt and tangent spaces. The feature pyramid size depends on the texture measure used and their parameter settings. SVM Ranking is used to reduce the number of features for all the descriptor to 64. A more detailed presentation of the experimental procedure and data set are given in Section 4.

### 3.4 Proposed Method I: Rotation fields pyramid

The first proposed new approach is based on multi‐resolution rotation fields. Rotation Fields are a very good means of capturing high frequency information from surface orientation. Nehab et al employed these to correct the three‐dimensional position of 3D mesh vertices with accurate high frequency data from normal maps captured with photometric stereo.^38^ Frequency separation has been extensively used in the literature to represent two‐dimensional texture.^39^, ^40^ This generally involves a pyramidal multi‐resolution representation, which allows the capture of texture information at different scales. At each level of the pyramid, the low frequency information is separated from the high frequency; the former is related to global shape, and the latter can be a good representation of local texture. We propose a multi‐resolution analysis scheme, where at each level of the pyramid the low frequency information in the normal map is separated from the high frequency in the form of rotation fields.

#### 3.4.1 Rotation fields

Let N denote a normal map and $N_{i,j}$, the normal vector at the pixel $p_{i,j}$. A smoothed version $N_{s}$ of N is found by computing at each pixel either a weighted geodesic or Euclidean mean over a neighbourhood with a radius r. A post‐normalisation of the resulting normal is required in the case of the Euclidean mean. The weights $w_{i,j}$ are determined by a Gaussian with a same radius r as the neighbourhood. The geodesic mean is defined as:

(5) $$\mu ={\text{argmin}}_{N^{\prime }}\sum_{p_{i,j}\in \Omega }d\left(N_{i,j},N^{\prime }\right)$$

With $d\left(N_{i,j},N^{\prime }\right)$ the geodesic distance between $N_{i,j}$ and $N^{\prime }$. Pennec^41^ show that this can be recursively approximated by:

(6) $$\mu^{t+1}=Exp_{\mu^{t}}\left(\frac{1}{card\left(\Omega \right)}\sum_{\Omega }\log_{\mu^{t}}\left(N\left(i,j\right)\right)\right)$$

Introducing the Gaussian weights $w_{i,j}$, gives:

(7) $$\mu^{t+1}=Exp_{\mu^{t}}\left(\frac{1}{card\left(\Omega \right)}\sum_{\Omega }w_{i,j}\log_{\mu^{t}}\left(N\left(i,j\right)\right)\right)$$

where $Exp_{\mu^{t}}$ and $\log_{\mu^{t}}$ are the exponential and logarithm map about the geodesic mean $\mu^{t}$. The rotation field R is obtained by computing the rotation to apply to the original normals to match the smoothed ones at each pixel. An axis‐angle representation $\left[\vec{e},\theta \right]$ can be adopted to characterise each rotation with four parameters (three for the axis $\vec{e}$ and one for the angle θ). Denoting $R^{\vec{e}}$ as the axis component of the rotation field and $R^{\theta }$ the angle component, we obtain:

(8) $$R_{i,j}^{\vec{e}}=N_{i,j}\times N_{i,j}^{\prime }\;\text{and}\;R_{i,j}^{\theta }=N_{i,j}\cdot N_{i,j}^{\prime }$$

The rotation axis $R^{\vec{e}}$ can be normalised to a unit vector so the rotation parameters can be brought down to three:

(9) $$R_{i,j}=\frac{R_{i,j}^{\vec{e}}}{\left||R_{i,j}^{\vec{e}}|\right|}R_{i,j}^{\theta }$$

**Figure 2:**
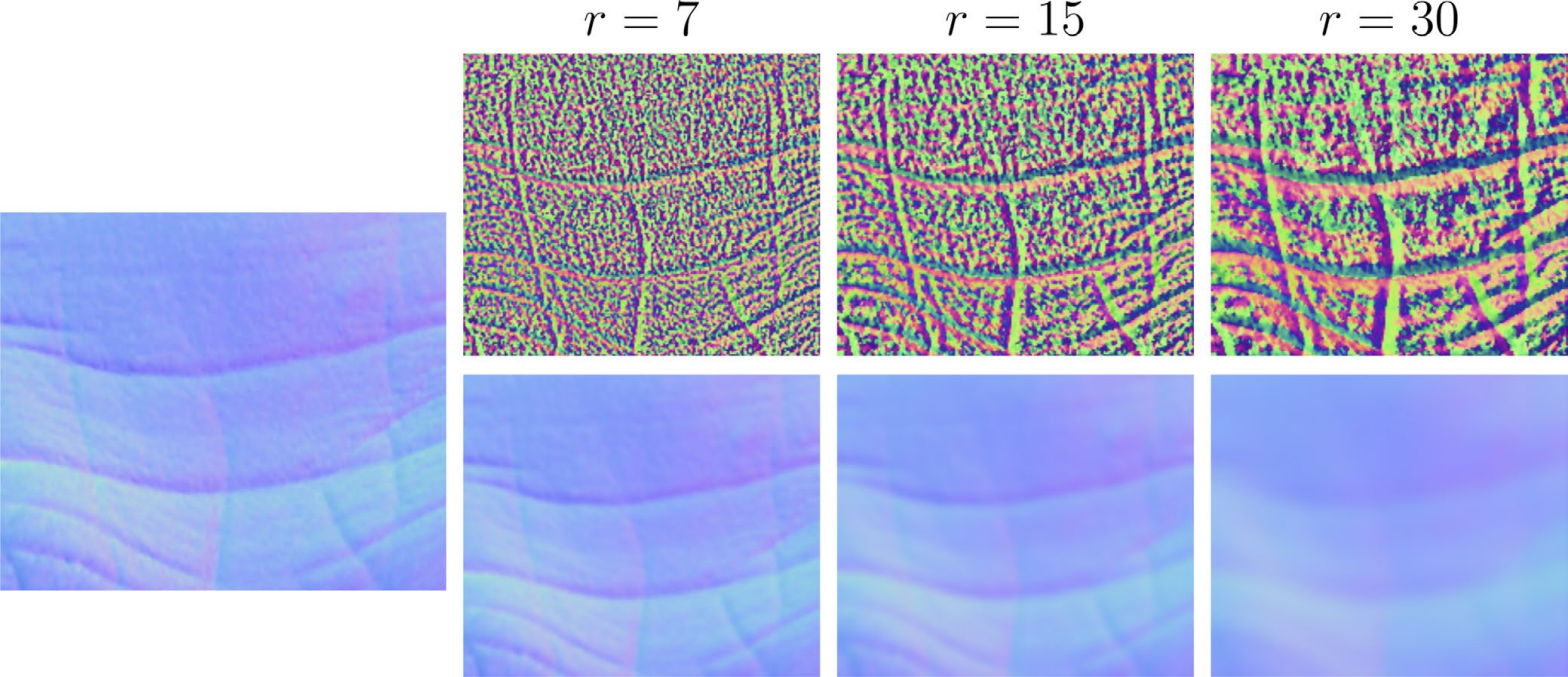
Rotation maps (top row) and corresponding low frequencies (bottom row) with different radius values (r=7,15,30) of a wrinkly normal map patch [Colour figure can be viewed at http://www.wileyonlinelibrary.com]

For visualisation purposes, these three parameters are encoded in an RGB image. The smoothing radius r controls the level of detail extracted. Small values of r allow the extraction of very fine skin texture (down to the level of pores) while higher values tend to capture medium frequency structures such as acne and wrinkles. Figure 2 shows the rotation maps and corresponding low frequencies of a wrinkly normal map patch computed with three different radius values.

### 3.5 Rotation fields pyramid

Given a normal map N, we perform a sub‐band decomposition by building an image pyramid where at each level, the low frequency information is separated from the high frequency. The initial step applies a low pass filter $G_{0}$, namely a Gaussian (with geodesic or Euclidean averaging). The result is a normal map $L_{0}$ representing the low frequency surface variation of the original one. Then, the high frequency information is extracted by calculating the rotation field that brings it back to the original normal map. After extracting the high frequency in the form of rotation field $H_{0}$, the low frequency normal map $L_{0}$ is then down‐sampled and passed on to the next level where the same process is repeated.

**Figure 3:**
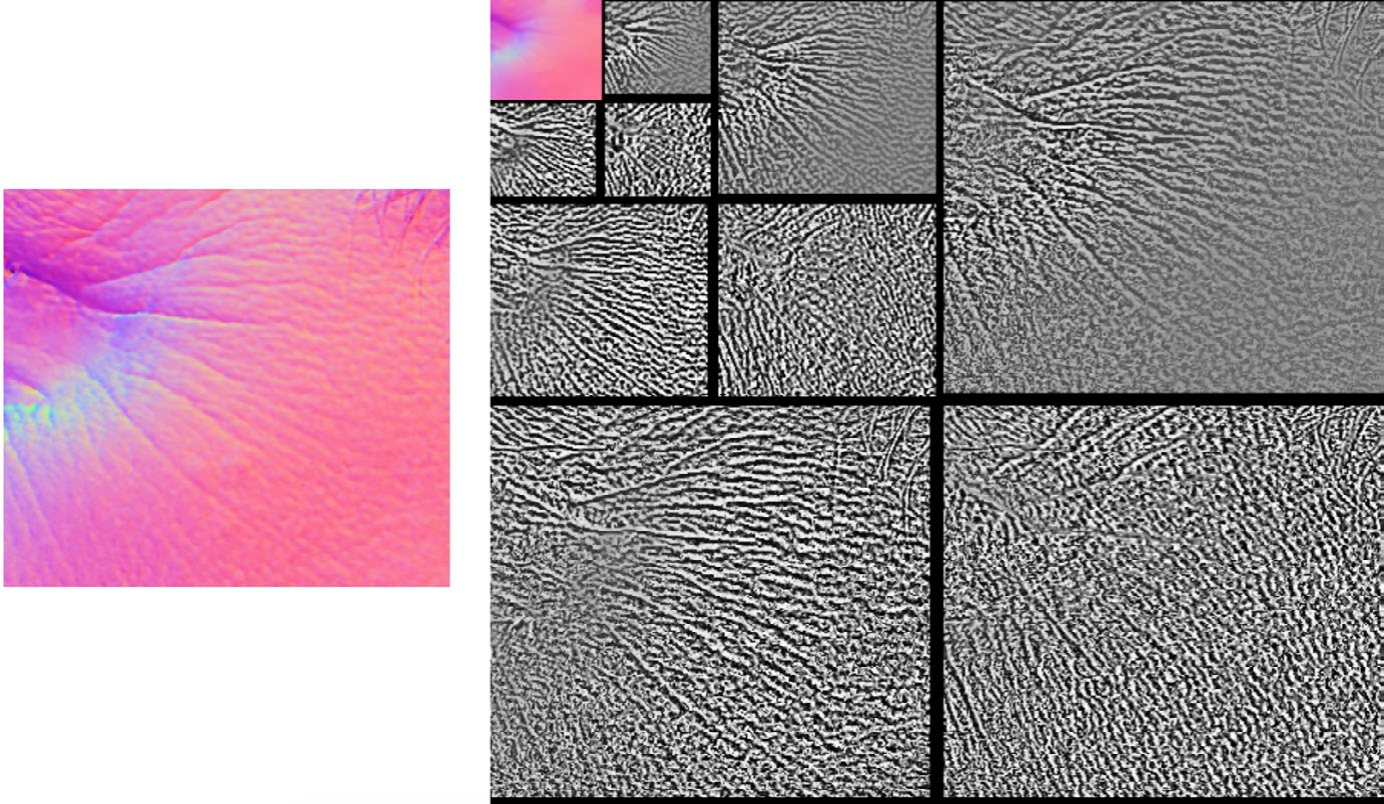
A 3‐level Rotation Fields Pyramid of a Wrinkly Normal Map Patch [Colour figure can be viewed at http://www.wileyonlinelibrary.com]

In the two‐dimensional case, most of the studies that use a pyramidal representation extract the high frequency information in several sub‐bands. The main motivation for this is to capture different spatial configurations and orientations of the texture. For example, Heeger and Bergen^39^ employed steerable filters to capture anisotropic texture with the presence of elongated or oriented structures. However, in contrast to individual pixels in a 2D image, each surface orientation in the normal map encodes information about the surface gradient within its immediate neighbourhood. So, at each level of the pyramid, we use three sub‐bands that correspond to the three components of the rotation vector, respectively. Figure 3 shows a 3‐level rotation field pyramid of a wrinkly normal map patch.

#### 3.5.1 Riemannian distance on the rotations group ${\text{SO}}_{3}$

After having represented the three‐dimensional surface texture as an n‐level pyramid of rotation fields, a metric is needed in the rotation space in order to analyse their spatial distribution. This problem has been well studied by Pennec^42^ Rotations can be represented not only by axis‐angle, but also by 3×3 orthogonal matrices which form the Rotation Group $SO_{3}$ and constitute a smooth manifold.^42^ This means that the set of rotation matrices is differentiable and support a Riemannian metric allowing to compute distances between rotations. If $R_{1}$ and $R_{2}$ are two rotation matrices and $R_{1}$ and $R_{2}$, respectively, the corresponding axis‐angle representations (the conversion can be easily done with the Rodriguez formula), the Riemannian distance between $R_{1}$ and $R_{2}$ is given by^42^:

(10) $$d:\begin{array}{c} {\text{SO}}_{3}\times {\text{SO}}_{3}\to R\\ \left(R_{1},R_{2}\right)\to d\left(R_{1},R_{2}\right)=\left(R_{2}\circ R_{1}\right) \end{array}$$

Although the composition of rotations can be calculated by the dot product of the two matrices $\left(R_{2}\circ R_{1}∼R_{2}^{T}R_{1}\right)$, Pennec^42^ showed that it is more advantageous to use unit quaternions as an intermediate step because the result is easier to differentiate. The idea is to convert the axis‐angle representation of the rotations to unit quaternions, multiply these and convert back into axis‐angle representation. Let R be an axis‐angle rotation (axis denoted by $R^{\vec{e}}$ and angle by $R^{\theta }$ and its corresponding unit quaternion Q represented by its scalar s and vectorial v parts, the conversions are given by:

(11) $$Q:\left\{\begin{array}{c} s=\cos\frac{R^{\theta }}{2}\\ v=R^{\vec{e}}\sin\frac{R^{\theta }}{2} \end{array}\right.\text{and}\;R:\left\{\begin{array}{c} R=2a\tan2\left(\left|v\right|,s\right)\\ R^{\vec{e}}=\frac{v}{\sin\frac{\theta }{2}} \end{array}\right.$$

And for two unit quaternions $Q_{1}\left(s_{1},v_{1}\right)$ and $Q_{2}\left(s_{2},v_{2}\right)$, the non‐commutative multiplication is given by:

(12) $$Q_{1}*Q_{2}:\left\{\begin{array}{c} s_{1}s_{2}-v_{1}\cdot v_{2}\\ s_{1}v_{2}+s_{2}v_{1}+v_{1}\times v_{2} \end{array}\right.$$

Equations 10, 11 and 12 give:

(13) $$d\left(R_{1},R_{2}\right)=\text{atan}2\left(\left|s_{1}v_{2}+s_{2}v_{1}+v_{1}\times v_{2}\right|,s_{1}s_{2}-v_{1}v_{2}\right)$$

Replacing s and v from equation 11 in equation 13 yields:

(14) $$d\left(R_{1},R_{2}\right)=a\tan2\left(\begin{array}{c} \left|R_{2}^{\vec{e}}\cos\frac{R_{1}^{\theta }}{2}\sin\frac{R_{2}^{\theta }}{2}+R_{1}^{\vec{e}}\cos\frac{R_{2}^{\theta }}{2}\sin\frac{R_{1}^{\theta }}{2}+\left(R_{1}^{\vec{e}}\times R_{2}^{\vec{e}}\right)\sin\frac{R_{1}^{\theta }}{2}\cos\frac{R_{2}^{\theta }}{2}\right|,\\ \cos\frac{R_{1}^{\theta }}{2}\sin\frac{R_{2}^{\theta }}{2}-\left(R_{1}^{\vec{e}}\cdot R_{2}^{\vec{e}}\right)\sin\frac{R_{1}^{\theta }}{2}\cos\frac{R_{2}^{\theta }}{2} \end{array}\right)$$

For the sake of simplicity, we assume that the two rotation axes $R_{1}^{\vec{e}}$ and $R_{2}^{\vec{e}}$ are parallel. This simplifies equation 14 to:

(15) $$d\left(R_{1},R_{2}\right)=a\tan2\left(\begin{array}{c} \sin\left(\frac{R_{1}^{\theta }}{2}+\frac{R_{2}^{\theta }}{2}\right),\\ \cos\left(\frac{R_{1}^{\theta }}{2}+\frac{R_{2}^{\theta }}{2}\right), \end{array}\right)$$

In our application, this simplification does not alter the captured information in terms of surface irregularities. Indeed, on the rotation map, each pixel represents the rotation of the original surface normal from the smooth one and hence is characterised by two parameters: the axis and the angle of rotation. The angle quantifies how much the two normals deviate from each other whereas the axis determines the plane in which the rotation happens. Now when we compute the distance between two rotations, we are more interested in capturing the deviation component than the orientation component of the rotation. This leads us to assume that the two rotations have the same axis which considerably simplifies the calculation without losing the deviation information we want to capture.

#### 3.5.2 Feature extraction and classification

For a given Normal Map patch, we compute an l‐level rotation field pyramid. For each level of the pyramid, we compute at each pixel the distances between the corresponding rotation and each of the neighbouring pixels within a N×M neighbourhood using Equation 15. This gives a vector of length N×M-1 at each pixel. We complete this vector with the rotation vector at the central pixel yielding then a vector N×M+2 long. A vector quantisation algorithm is used to map each of these N×M+2 vectors to a scalar value. In this work, we use K‐means which introduces another parameter k representing the number of clusters. Each cluster is associated with a symbolic label (a scalar value). We then compute the histogram of the resulting map of symbolic labels. The size of the histogram is given by the number of clusters k. The process is repeated at each level in the pyramid, and the histograms from all the levels are concatenated to form the l×k feature vector associated with the patch.

The method is tested on classifying the three skin conditions from our collected 3D facial data set. We experimented with two different K‐means configurations, k=100 and k=200, yielding, respectively, with a 3‐level pyramid, 300 and 600 long feature vectors. SVM ranking is then used to reduce both feature vectors to 64 components. Also, various sample sizes were tried: 20×20 pixels, 50×50 pixels and 80×80 pixels. Section 4 gives a more detailed presentation of the data set and experimental set‐up.

### 3.6 Proposed new method II: Local orientation patterns

The second approach we propose for analysing 3D surface texture from normal maps is based on the generalised Texture Spectrum,^43^ introduced by Wang and He, and defined as the distribution of texture entities called Texture Units over an image. In the original formulation, a Texture Unit is a 3×3 pixel neighbourhood forming a window of 8 pixels ${\left(p_{i}\right)}_{1\leq i\leq 8}$ surrounding a central one $p_{0}$. Each of the 8 surrounding pixels may be associated with 3 possible patterns defined by the function ${\left(f_{i}\right)}_{1\leq i\leq 8}$:

(16) $${\left(f_{i}\right)}_{1\leq i\leq 8}=\left\{\begin{array}{cc} 0 & \text{if}\;p_{i}<p_{0}\\ 1 & \text{if}\;p_{i}=p_{0}\\ 2 & \text{if}\;p_{i}>p_{0} \end{array}\right.$$

The value of the Texture Unit associated to $p_{0}$ is determined from the 8 surrounding patterns by:

(17) $$f\left(p_{0}\right)=\sum_{i=1}^{8}f_{i}\times 3^{i-1}$$

The notion of Texture Spectrum can be generalised by extending the definition of a Texture Unit to n possible patterns between two pixels and an arbitrary number of N pixels uniformly surrounding a central pixel $p_{0}$ with an arbitrary radius of r. In these cases, the Texture Unit function (Equation 17) becomes:

(18) $$f\left(p_{0}\right)=\sum_{i=1}^{N}f_{i}\times n^{i-1}$$

The patterns ${\left(f_{i}\right)}_{1\leq i\leq N}$ can be defined with any discrete two‐dimensional function that has only n possible values in Z+.

A Texture Unit is associated with each pixel contained in the image, and the Texture Spectrum is defined as the distribution of Texture Units over the whole image. This is represented by a histogram counting the frequency of each possible Texture Unit value over the image.

The main task here is to find good pattern functions that can represent the normals’ orientation distribution over a Texture Unit. We propose two pattern functions for representing the normals’ orientation distribution. The first function computes the dot product of two normals and compares the result with a threshold. The second function compares the azimuthal and polar angles of the normals directly.

#### 3.6.1 1st pattern function

The first pattern function we propose evaluates the dot product between the central normal and one of the surrounding normals, and compares the result to a threshold. Formally, it is given by (with a threshold τ):

(19) $$f_{i}^{\tau }\left(N_{0},N_{i}\right)=\left\{\begin{array}{cc} 0 & \text{if}\;N_{0}\cdot N_{i}<\tau \\ 1 & \text{if}\;N_{0}\cdot N_{i}\geq \tau \end{array}\right.$$

With this pattern function, the number of bins needed for the histogram is given by $2^{N}$ as in Local Binary Patterns. As the normals are normalised in $\left[-1,1\right]$, the dot product depends only on the angle between the two normals. However, the problem here is to find a good threshold. It is clear that a good threshold depends on the local orientation distributions in the normal map; a good threshold for a dense and/or more or less uniform normal map may not be suitable for a sparser normal map. The threshold choice also depends on the application; for the same normal map, we may use different thresholds depending on whether we want to capture high or low frequency variations (although this would need to be combined with an adequate radius setting).

**Figure 4:**
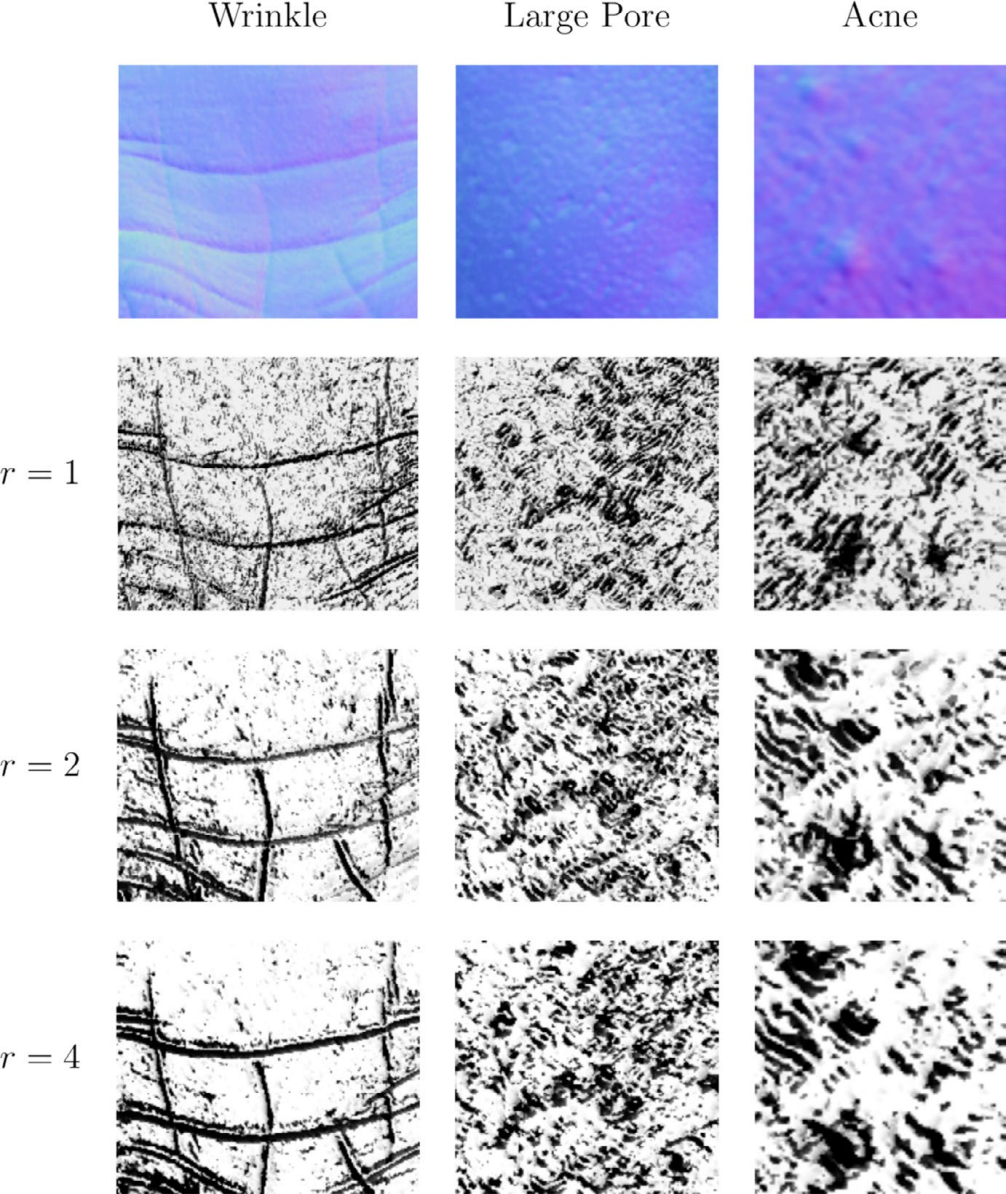
Local Orientation Pattern of skin normal maps with different radius using the first pattern function [Colour figure can be viewed at http://www.wileyonlinelibrary.com]

We have tried two techniques for choosing the threshold. The first averages the dot products of all pairs of normals. The second method computes a threshold map by locally averaging the dot products between each normal in a Texture Unit with the central normal. Our experiments show that the first method achieves better results than the second, although a good threshold map may provide additional robustness in cases where the distribution of the normal orientations varies considerably from one place to another. Figure 4 shows the Local Orientation Pattern Images of three skin patches using the first pattern function with a radius of 1, 2 and 4.

#### 3.6.2 2nd pattern function

In the second proposed pattern function, the azimuthal and polar angles of the normal are compared directly. The function has four possible values and is defined by:

(20) $$f_{i}\left(N_{0},N_{i}\right)=\left\{\begin{array}{cc} 0 & \text{if}\;\theta_{0}<\theta_{i}\;\text{and}\;\phi_{0}<\phi_{i}\\ 1 & \text{if}\;\theta_{0}<\theta_{i}\;\text{and}\;\phi_{0}\geq \phi_{i}\\ 2 & \text{if}\;\theta_{0}\geq \theta_{i}\;\text{and}\;\phi_{0}<\phi_{i}\\ 3 & \text{if}\;\theta_{0}\geq \theta_{i}\;\text{and}\;\phi_{0}\geq \phi_{i} \end{array}\right.$$

**Figure 5:**
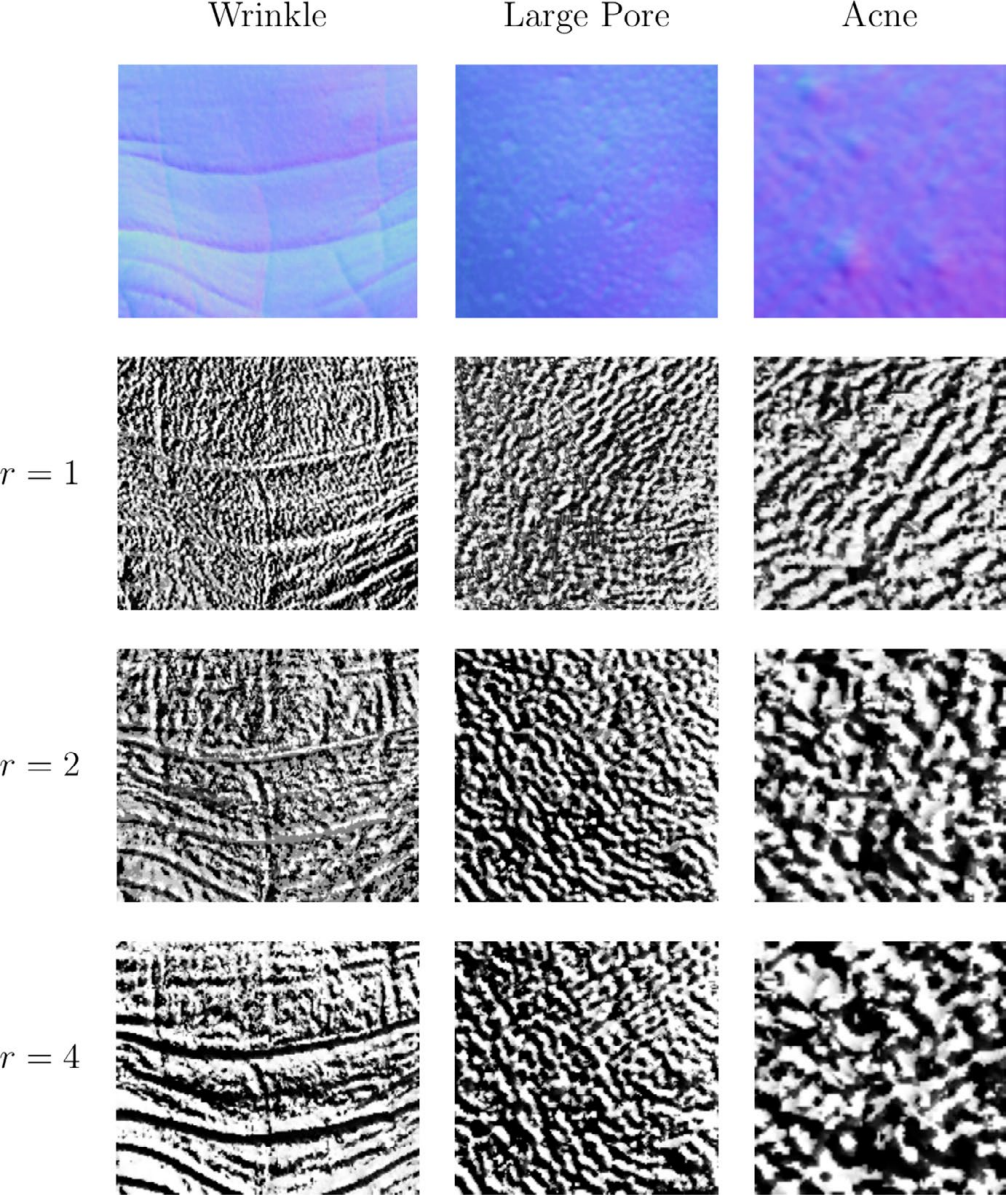
Local Orientation Pattern of skin normal maps with different radius, using the second pattern function [Colour figure can be viewed at http://www.wileyonlinelibrary.com]

$\theta_{i}$ and $\phi_{i}$ are, respectively, the azimuthal and polar angle of the normal $N_{i}$. Here, the required size of the histogram is given by $4^{N}$. This function does not need the extra threshold parameter that the first one does, although it generates a much bigger feature vector. While the first function generates (for the standard 8‐pixel neighbourhood) a feature vector of length 256, this function generates a 65536‐element feature vector. Figure 5 shows the Local Orientation Pattern Images of three skin patches using the second pattern function with a radius of 1, 2 and 4. The visualisations are produced by converting the binary pattern at each pixel to a scalar value.

#### 3.6.3 Feature extraction and classification

A glance at the LOP (Local Orientation Patterns) images in Figure 4 and Figure 5 gives a first idea of the behaviour difference between the two proposed pattern functions. The second pattern function tends to produce LOP images with higher frequency. This is probably due to the level of detail generated by using four patterns instead of just two. The important point here is that when using the second pattern function for capturing low frequency properties of a surface, a certain amount of noise, depending on how fine the surface structure is, can be detected. In our applications, we think that it is more appropriate to use this second function for high frequency skin properties such as pores and some lines and wrinkles, while the first function is more appropriate for capturing lower frequency conditions such as acne.

### 3.7 Proposed Method III: Multi‐scale Azimuthal projection distance

The third novel method we propose is an extension of the Azimuthal Projection Distance Image (APDI) introduced by Sandbach et al as a 3D surface descriptor for facial Action Unit detection.^28^ In their work, the authors used the APDI for coarse scale and extracted facial macro‐structure. However, while these facial macro‐structures are adequate for discriminating Action Units, they do not hold enough surface fine‐scale detail to accurately characterise the skin conditions we are interested in (wrinkles, large pore and acne). We thus extend the APDI with three main additions:

- We work with local surface normal means instead of a fixed surface mean as reference for the azimuthal projection.
- We have modified the APDI formula to take into account the surface normal azimuthal orientation, which is not considered in the original formulation.
- We have introduced a multi‐resolution analysis scheme in order to capture different scales of skin deformations.

In the original formulation,^28^ the APDI is a 2D image where the pixels are the projections of the surface normals onto the tangent plane. Given a surface normal at a pixel $\left(i,j\right)$, the azimuthal projection is given by:

(21) $$\begin{array}{ccc} x_{i,j} & = & k^{\prime }\cos\theta_{i,j}\sin\left[\phi_{i,j}-\bar{\phi }i,j\right]\\ y_{i,j} & = & k^{\prime }\left\{\cos{\bar{\theta }}_{i,j}\sin\phi_{i,j}-\sin{\bar{\theta }}_{i,j}\cos\theta_{i,j}\cos\left[\phi_{i,j}-{\bar{\phi }}_{i,j}\right]\right\} \end{array}$$

where $\theta_{i,j}$ and $\phi_{i,j}$ are the polar and azimuthal angles of the surface normal, respectively, and ${\bar{\theta }}_{i,j}$ and ${\bar{\phi }}_{i,j}$ are the polar and azimuthal angles of the mean surface normal over a fixed neighbourhood around $\left(i,j\right)$, respectively. Finally, $k^{\prime }=\frac{c}{\sin\left(c\right)}$ with $c=\sin{\bar{\theta }}_{i,j}\sin\theta_{i,j}+\cos\theta_{i,j}\cos\left[\phi_{i,j}-{\bar{\phi }}_{i,j}\right]$.

Sandbach et al fixed a constant mean surface normal $\left(0,0,1\right)$ (z‐axis direction) which leads to $\bar{\theta }=\frac{\pi }{2}$, $\bar{\phi }=0$ and $c=\sin\theta_{i,j}$. Thus equation 21 become:

(22) $$\begin{array}{ccc} x_{i,j} & = & k^{\prime }\cos\theta_{i,j}\sin\phi_{i,j}\\ y_{i,j} & = & k^{\prime }\cos\theta_{i,j}\cos\phi_{i,j} \end{array}$$

Each pixel value of the APDI is given by the $L^{2}$ norm of $\left(x_{i,j},y_{i,j}\right)$:

(23) $$APDI_{i,j}=\sqrt{x_{i,j}^{2}+y_{i,j}^{2}}$$

#### 3.7.1 Modified APDI

As stated above, in the original formulation, the authors set a constant surface normal mean $\left[0,0,1\right]$ over the whole face, thus projecting about a constant vector across the face. A direct consequence of this is the presence of considerable low frequency information in the APDI, as the mean surface normal constitutes the reference about which the normals are projected (the tangent plane that the normal are projected onto is the plane orthogonal to the mean surface normal). While this is suitable for coarse features such as facial Action Units, it would introduce notable low frequency bias to the fine skin structures we are interested in. Thus, we compute at each pixel a local mean surface normal over a specified neighbourhood and use it as projection reference. Hence, in this work we use equation 21 instead of the simplified versions of Sandbach et al

**Figure 6:**
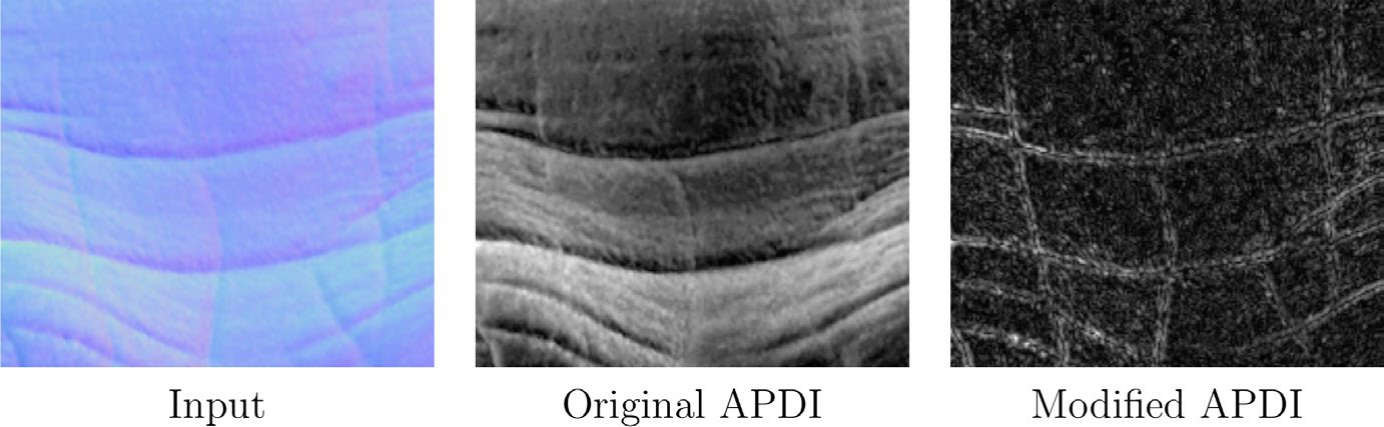
Example of output from the original and modified APDI [Colour figure can be viewed at http://www.wileyonlinelibrary.com]

Figure 6 shows some example outputs from the original and our proposed modified APDI. On the output image from the original APDI, the low frequency is still very noticeable whereas in the modified version only the high frequency information is kept.

**Figure 7:**
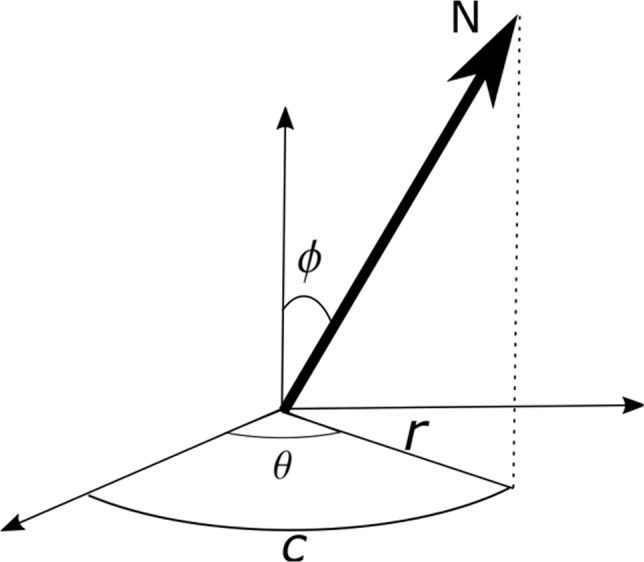
The distance from the centre of projection r is the same for all the normals with the same polar angle, while the arc c from the X- axis varies with both the azimuthal and polar angles

Another modification made to the original APDI formulation is the introduction of the azimuthal orientation of the surface normal in Equation 23 which only takes into account the polar orientation. This is illustrated in Figure 7, where the mean surface normal is assumed to be aligned to the z‐axis. It is easy to see that the distance r from the centre of projection, which corresponds to the original formula, stays constant for all normals with the same polar angle ϕ even though the azimuthal angle θ varies. This is overcome by changing Equation 23 to:

(24) $$APDI_{i,j}^{m}=\arctan2\left(x_{i,j},y_{i,j}\right)\sqrt{x_{i,j}^{2}+y_{i,j}^{2}}$$

This corresponds to the arc c in the projection plane going from the x‐axis to the projected point and varies with ϕ as well as θ.

**Figure 8:**
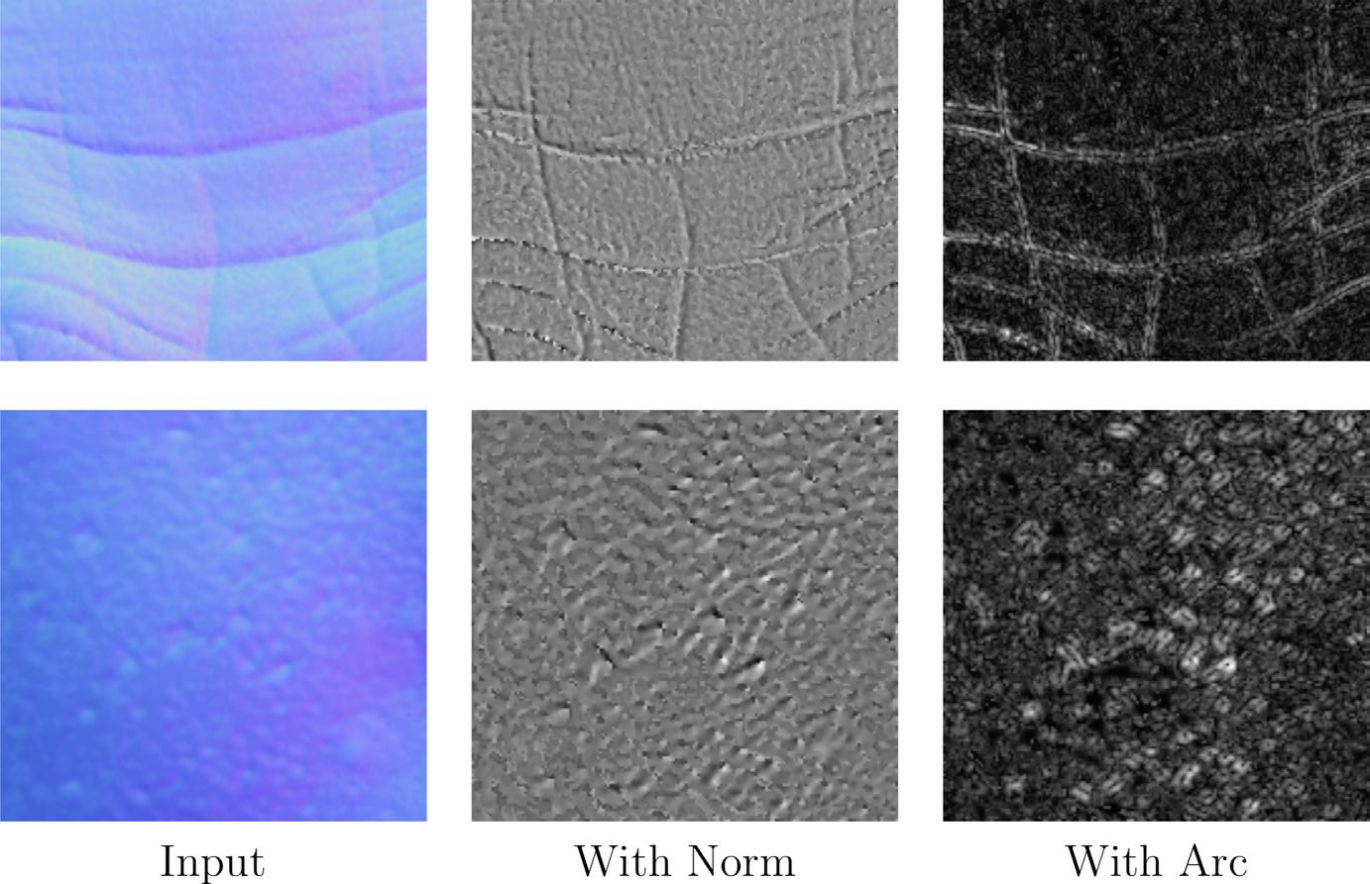
Example of output from considering the $L^{2}$ norm (original) and the arc (modified) [Colour figure can be viewed at http://www.wileyonlinelibrary.com]

Figure 8 shows the difference between using the $L^{2}$ norm (distance from the centre of projection) or the arc from the X‐axis. In the first case ($L^{2}$ norm), the APDI appears less contrasted in comparison with the second case (arc) which presents more disparity and hence will be more discriminative as shown in the classification results in Section 4.

#### 3.7.2 Multi‐resolution scheme

We employ a multi‐scale APDI scheme for analysing the 3D skin texture from dense surface orientations. For a given normal map, a multi‐scale APDI pyramid is built by computing the normal map's APDIs at different resolutions. This involves scaling (down‐ or up‐sampling) the normal map. Since the surface normals do not satisfy the linearity condition required by classical convolution methods, we use a geodesic‐based normal map scaling algorithm.

We use Riemannian differential geometry elements to introduce a new metric (geodesic distance) which will allow us to perform linear operations on the normals. We assume the normals to be on a Riemannian manifold and compute all linear operations on a tangent plane that is chosen to be constant for all the normals.^42^ Let $Exp_{\mu }$ and $Log_{\mu }$ be the Riemannian Exponential and Logarithm operations with μ as projection axis, the linear combination of N normals ${\left(ni\right)}_{1\leq i\leq N}$ with coefficients ${\left(\alpha \right)}_{1\leq i\leq N}$ can be computed as:

(25) $$f\left(n_{i},\alpha_{i}\right)=\exp_{\mu }\left(\sum_{i=1}^{N}\alpha_{i}\times {\text{Log}}_{\mu }\right)$$

By the definition of the exponential mapping, the result will always be a unit vector. Our scaling algorithm is based on Equation 25. As we are only interested in down‐sampling, we present an overview of the down‐sampling algorithm below.

#### 3.7.3 Algorithm 1: Normal map down‐sampling algorithm

Inputs: normal map N, scale factor S, window size [u,v]
n_w_ = width(N)/S
n_h_ = height(N)/S
for i=1 to n_w_
 for j=1 to n_h_
 tmp = 0
 w = i-u/2
 for k=1 to u
 y = j-v/2
 for l = 1 to v
 tmp = tmp + Log_μ_(N(w,y))
 y = y+1
 end
 w = w+1
 end
 M(i,j) = Exp_μ_(tmp/(u*v))
 end
end
Return: down-sampled normal map M

The full implementation includes border checking and index checking which has been omitted here for brevity. We have tested the proposed method by comparing a normal map with the result of down‐sampling and up‐sampling it back. The geodesic method achieves 0.027 mean angular error, while using a classical sampling method on each channel and renormalising back the result gives a mean angular error of 0.183.

**Figure 9:**
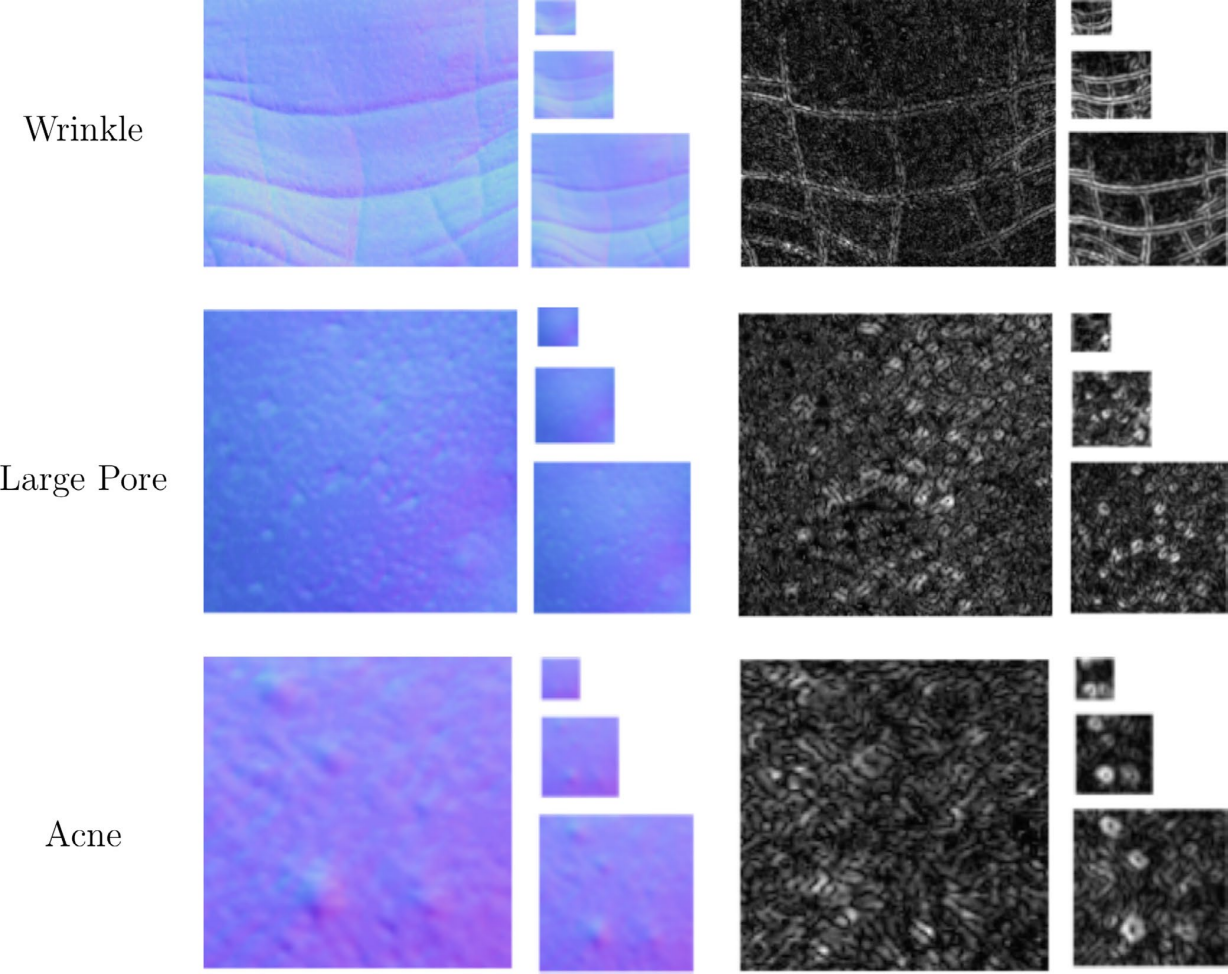
A four level APDI Pyramid for 3 Skin Patches [Colour figure can be viewed at http://www.wileyonlinelibrary.com]

To characterise the 3D skin texture, we build a multi‐resolution pyramid of APDIs by down‐sampling the normal map to different levels. At each level, the APDI is re‐computed from the corresponding down‐sampled normal map. The high levels contain higher frequency details adequate for texture analysis. The lower levels lose high frequency detail, but the low frequency changes related to the overall shape are highlighted. Figure 9 shows examples of image output of the modified multi‐resolution APDI for 3 skin patches with presence of wrinkles, large pores and acne, respectively. It is interesting to notice how, at different scales, the level of high frequency information that is captured changes. For example, considering the patch with acne, one can see that on the first level, only the fine skin structure is captured. It is clear that stopping the texture extraction at that level would capture only partial information about the skin disruption and would certainly miss the big skin spots. These are captured better by the subsequent levels as shown Figure 9.

#### 3.7.4 Feature extraction and classification

To extract features from a given normal map patch, the multi‐resolution APDI pyramid is built. Then, a grey level histogram is computed at each level of the pyramid and concatenated together. This produces a relatively big feature vector depending on the number of levels and the histogram resolution (eg number of bins). For example, an 128‐bin histogram with a 4‐level pyramid will produce a feature vector of length 512. This can be reduced using feature selection techniques.

## 4 EXPERIMENTAL SET‐UP

### 4.1 Data set

The algorithms described in this paper are intended to work with data acquired in a light stage. A light stage is a 3D surface acquisition device first proposed by Debevec et al^31^ which is to date the most advanced set‐up for capturing surfaces’ fine structure. Existing 3D face data sets that use photometric stereo include the Photoface database 44 and the 3D Relightable Facial Expression (ICT‐3DRFE) database.^45^ While the first was captured with low‐cost cameras, the latter is captured using a light stage. Despite providing highly detailed 3D data, the ICT‐3DRFE database is not suitable for this work as the age range and skin types covered by the data set is limited.

To cover a wider age range and skin type, we have collected a new data set using our own light stage. The capture and processing of the acquired data are detailed here.^46^ Briefly, the data set comprises facial captures from 50 subjects ranging in age and skin condition. The subjects’ ages ranged from 19 to 68 years old. In addition to the facial images, various extra information about the subjects (age, sex, height, weight, eye colour, hair colour, makeup, ethnic origin etc) were collected. Male participants were more represented than female, with 41 men and 9 women. Various ethnic groups were represented, although the majority were Caucasian.

Each subject was captured from 3 directions (front, left and right), and the resulting textures were stitched together using a Poisson blending algorithm. The geometry is captured from 8 SLR cameras and is calculated across the 3 views, giving 24 images in total. 42 LEDs arranged on a geodesic dome are used to provide gradient pattern illumination, providing an additional 13 photometric images per view. Polarising filters are used on half of the photometric images in order to remove specular reflections. In total, 63 images are captured per subject and used to create the geometry, diffuse and specular texture maps, diffuse and specular normal maps.

#### 4.1.1 Region segmentation

**Figure 10:**
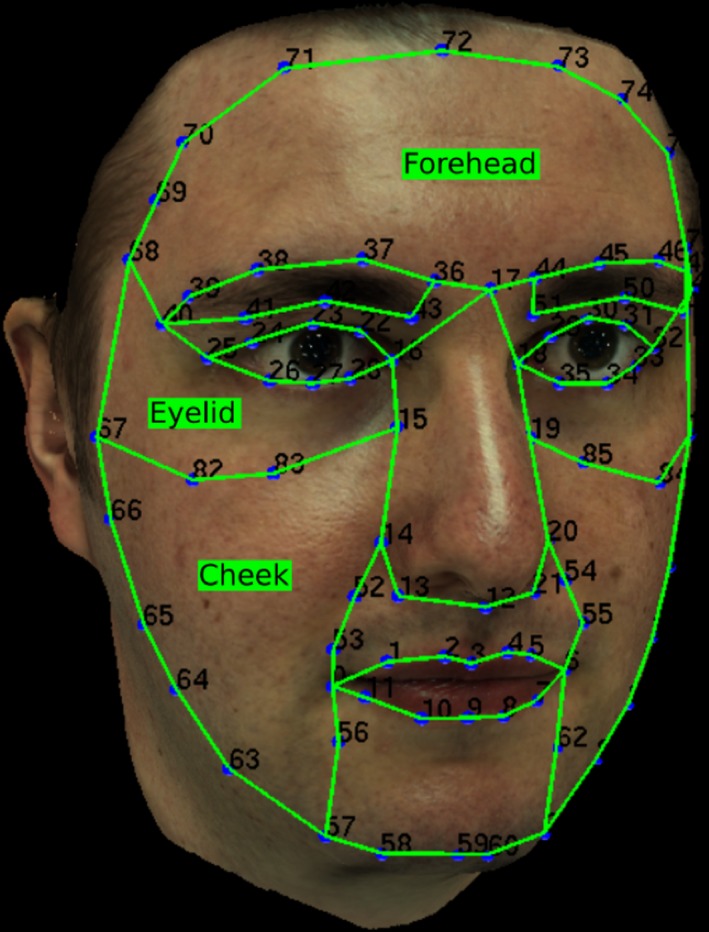
Template used to segment the captured face in regions of interest [Colour figure can be viewed at http://www.wileyonlinelibrary.com]

Each face was segmented into 14 regions using a 3D template (set of landmarks) manually adjusted on the face (Figure 10). As all processing (analysis or synthesis) is done on the measured normal maps, this segmentation is projected on the 2D texture space of each of the 3 photometric poses using the corresponding camera parameters.

**Figure 11:**
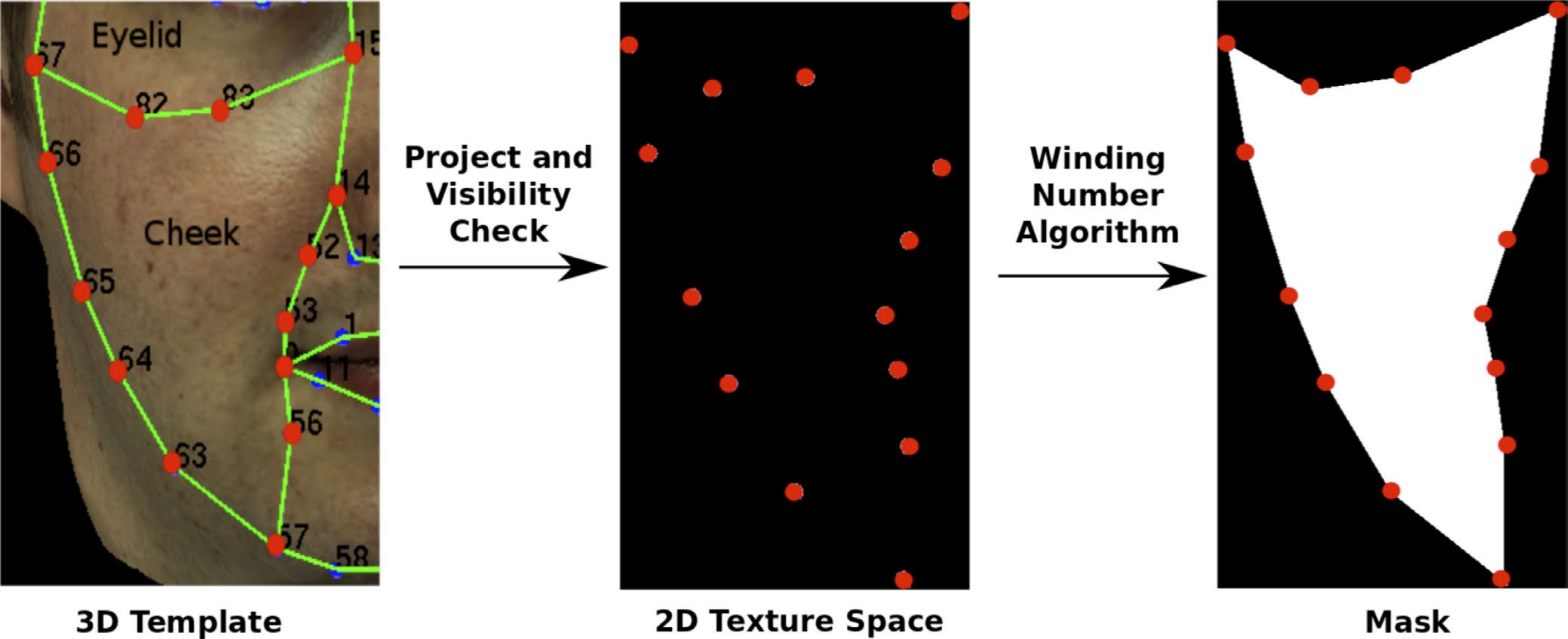
Example of region mask construction (left cheek on frontal pose) [Colour figure can be viewed at http://www.wileyonlinelibrary.com]

For each region, we first project its corresponding landmarks onto each of the poses using the camera parameters and a visibility calculation. This results in a set of 2D points in the texture space on each pose. We then use a winding number algorithm^47^ to compute the polygon formed by this set of points for each pose. This polygon is used as a mask for the corresponding region in a given pose. Figure 11 shows an example of region mask construction of the left cheek on the frontal pose.

### 4.2 Data annotation

**Figure 12:**
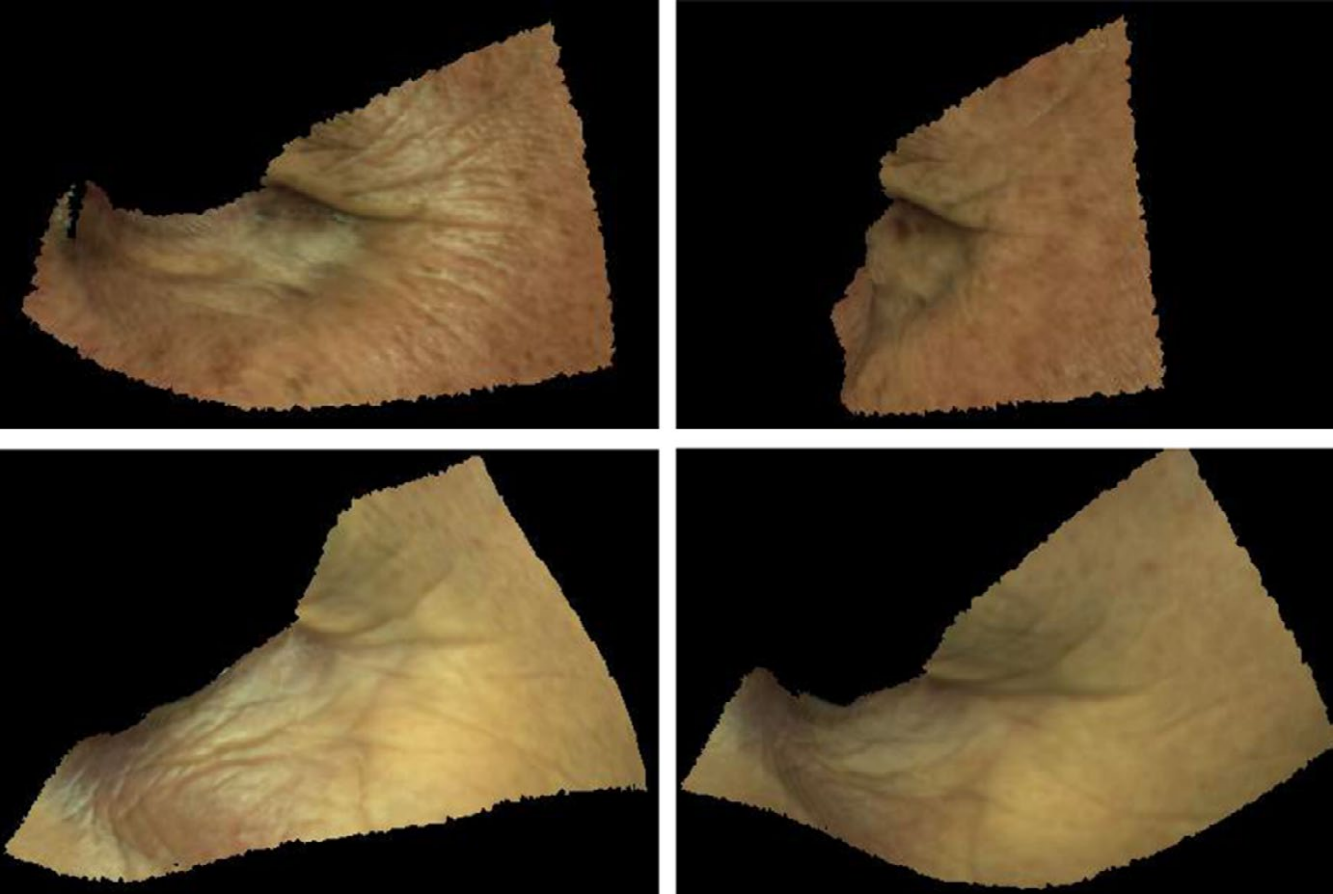
Change in apparent texture when viewpoint varies [Colour figure can be viewed at http://www.wileyonlinelibrary.com]

For data annotation, an experiment was conducted in which human participants were presented with skin patches from different regions of the face and asked to rate them on a scale of 1 to 5 according to the presence and visibility of wrinkles, acne and pores. We considered three regions of interest: cheek, forehead and eye corner, as these are the regions in which the skin conditions we are interested in occur most. All faces were segmented using the generic template shown in Figure 10. A photo‐realistic animation was rendered for each patch showing it at different angles with a fixed point light. The photo‐realism of this animation was critical to the rating process as the apparent texture of the skin is strongly affected by the lighting and viewing conditions. Figure 12 shows two skin patches rendered with two different viewing angles and the difference in apparent texture is clearly evident.

**Figure 13:**
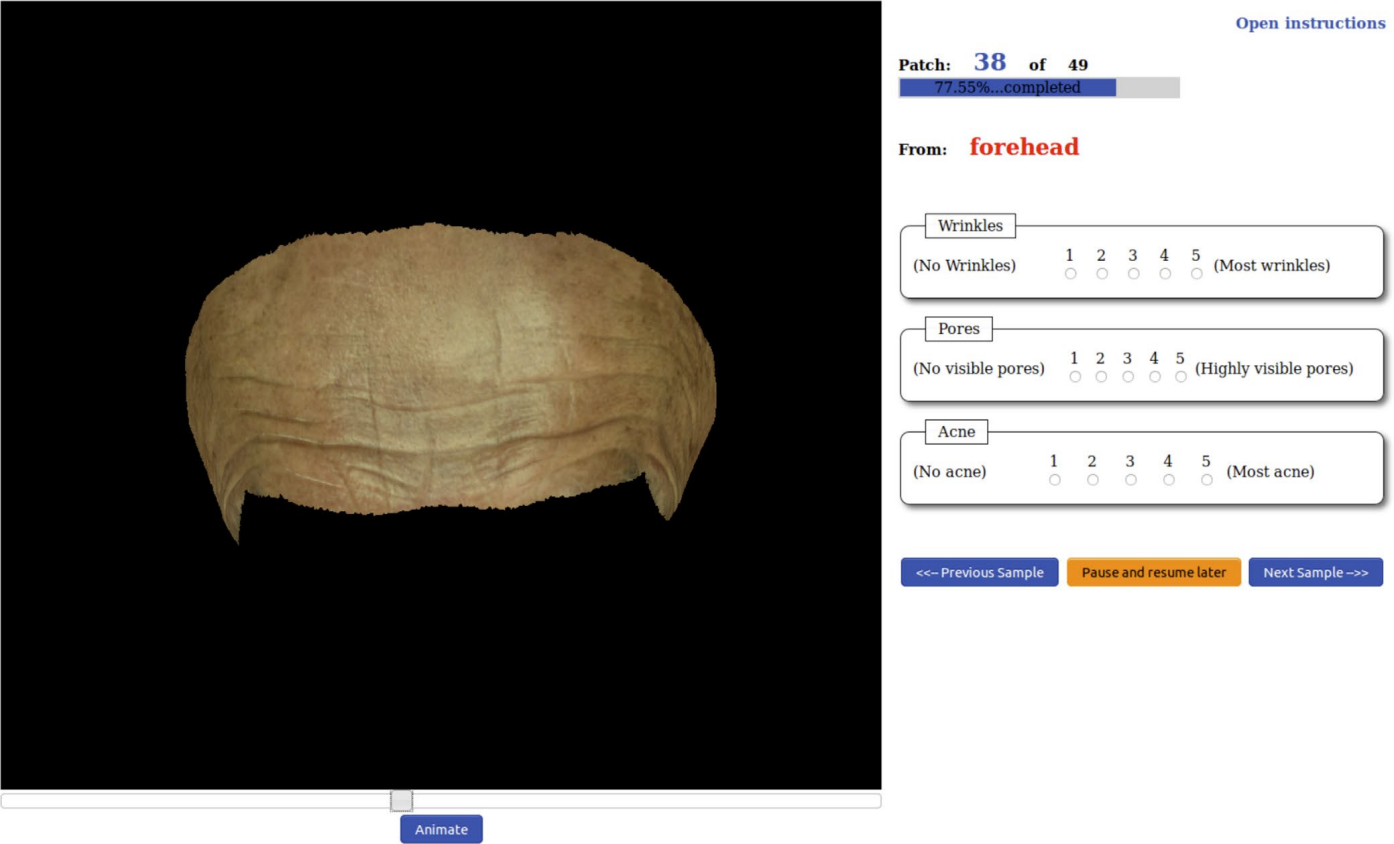
Rating Platform for the Psychophysical Experiment [Colour figure can be viewed at http://www.wileyonlinelibrary.com]

Our rating platform was set as a web application (Figure 13). The pre‐rendered animation of each skin patch was played to the participant at least once before any rating could be entered. The participant has the option to re‐run the animation as many times as they wish and to change the viewing angle manually using a slider control. To reduce potential ordering bias, the sequence allocated to each participant is randomised.

We assume that most of the skin conditions we are interested in are more or less symmetrical across the face (ie if a subject presents acne or large pores on the left cheek, it is likely that the same condition will be found on the right cheek). Thus, for each subject, instead of presenting both the left and right cheeks or eye corners to the raters, one side is picked randomly.

To reduce the rating time and minimise the risk of having participants withdraw before finishing a session, the patches were categorised in blocks according to their location on the face. Thus, we had three blocks (cheek, eyelid and forehead) of 50 patches each. A participant chose a block to start with, with the option of rating a second or third block upon completion.

Judgement of facial skin texture is a rather subjective task. The way people perceive and quantify the skin conditions that we are interested in will certainly be affected by many factors related to their own personal experience. Therefore, for our data set to be reliable, it was necessary to get it rated by many individuals. This also allowed analysis of the correlations between how different people perceive these skin conditions. A total of 25 participants rated the data set, with almost all of them having rated at least two blocks.

### 4.3 Inter‐rater agreement

As the data were rated by 25 participants, each sample has a set of ratings given by different individuals. Therefore, we can measure the data set's consistency by investigating agreement between ratings provided by different participants. Table 1 presents various correlation and agreement measures computed on the raw ratings. These show relatively low correlation and agreement between the raters on eye corner and forehead, but a strong agreement in the Cheek region.

**Table 1:** Various correlation and agreement measures on raw ratings

Region	Nb. Rater	Correlation		Agreement	
		Spearman's	Kendall's	Fleiss Kappa	Kripp alpha
Cheek	8	0.639	0.610	0.170	0.403
Eye corner	11	0.207	0.200	0.087	0.104
Forehead	8	0.273	0.260	0.074	0.073

The low correlation measures on the raw data suggest some disagreement between raters. This can be due to differences in judgement, raters not understanding the instructions, or raters not providing genuine ratings. To achieve higher inter‐rater agreement, we experimented with excluding those participants who correlate the least with the rest. Participants are excluded successively by ascending order of correlation to the rest, starting with the one with the weakest correlation value. However, excluding too many participants would result in decrease of confidence even though the apparent correlation obviously increases. Hence, the exclusion policy we used was as follow: we keep the maximum number of raters that achieves a correlation greater than or equal to 0.5.

## 5 RESULTS SUMMARY

We summarise here the classification results yielded with the 3D texture descriptors proposed in this paper. We also compare these against the performances of a BTF texton‐based method which, to date, is one of the most advanced ways used to represent illumination/view independent texture. We implemented the BTF texton‐based method by applying a bank of 14 filters (with six orientations, four differences of Gaussian and four Gaussian) to the collected specular intensity images. The filtering is done at three scales, which yields at each pixel a response vector of 42 elements. The input images are taken from three viewpoints for forehead patches, 2 viewpoints for cheek patches and all under seven different light directions, that is 14 or 21 images per patch. The resulting 882 (forehead) or 588 (cheek) responses per pixel per patch of all the images in the data set are then clustered using a K‐means algorithm, where k is fixed to 200. Each cluster is associated with a label that corresponds to a unique texton. The histogram of textons is then computed. This represents the feature vector associated with the corresponding sample.

In this work, we use the Weka implementation of the multi‐layer perceptron for training and classification, and we use a 10‐fold cross‐validation approach. This choice has been motivated by preliminary investigations with other classifiers including Random Forests and Support Vector Machine that both yielded poorer results. The number of network layers is set to Weka's default which is the mean of the number of classes and the number of attributes. The output of the classifier is a discrete rating of the presence or absence of the considered skin condition and, as defined in the ground truth, is a discrete number between “1” (meaning very low) and “5” (meaning very high). The results presented in Table 2 show the performances of each descriptor in terms of the F‐measure, which represents the harmonic mean of the precision and recall.

**Table 2:** Classification results

		Wrinkles				Pores				Acne		
		Sample size										
Features		20	50	80	20		50	80	20		50	80
2D R‐LBPs	Radius = 2	0.53	0.59	0.62	0.61		0.63	0.62	0.59		0.63	0.60
	Radius = 5	0.60	0.67	0.70	0.73		0.73	0.72	0.62		0.70	0.70
2D Gabor	Radius = 2	0.60	0.65	0.72	0.58		0.58	0.59	0.60		0.62	0.61
	Radius = 5	0.64	0.70	0.75	0.71		0.70	0.71	0.71		0.73	0.71
3D R‐LBPs	Slant/tilt	0.75	0.78	0.81	0.79		0.81	0.80	0.71		0.73	0.72
	Tangent	0.70	0.73	0.79	0.73		0.75	0.73	0.66		0.69	0.65
3D Gabor	Slant/tilt	0.78	0.80	0.82	0.83		0.85	0.85	0.75		0.76	0.77
	Tangent	0.74	0.78	0.81	0.77		0.79	0.79	0.70		0.74	0.72
APDI	—	0.62	0.61	0.65	0.63		0.60	0.62	0.60		0.63	0.63
M‐APDI	Depth = 1	0.62	0.65	0.68	0.62		0.62	0.64	0.61		0.65	0.64
	Depth = 2	0.69	0.70	0.73	0.71		0.70	0.72	0.68		0.67	0.70
	Depth = 4	0.75	0.78	0.81	0.74		0.76	0.73	0.72		0.74	0.75
BTF Texton	*K* = 100	0.81	0.85	0.88	0.85		0.86	0.86	0.86		0.89	0.88
	*K* = 200	**0.89**	**0.91**	**0.93**	0.87		**0.90**	0.86	**0.90**		**0.92**	**0.90**
LOP	1st PF	0.71	0.70	0.76	0.63		0.66	0.63	0.75		0.79	0.81
	2nd PF	0.72	0.72	0.77	0.79		0.81	0.80	0.73		0.78	0.83
Rot. Fields	*K* = 100	0.78	0.80	0.84	0.87		0.89	0.87	0.79		0.83	0.83
	*K* = 200	0.82	0.86	0.90	**0.91**		**0.90**	**0.92**	0.84		0.87	0.87

Bold values indicates the best performing method in each column

The overall results show that the 3D descriptors clearly outperform the 2D descriptors. First, on comparing R‐LBPs and Gabor filtering on 2D and 3D data, both texture characterisation methods show a clear improvement when used in a 3D configuration (slant/tilt or tangent space) for the classification of both wrinkles, acne and pores. The classification performances vary with the chosen patch size, which also seems to depend on the skin condition being classified. The results show that for all the descriptors the performance increases with the patch size when classifying wrinkles. However, this pattern does not seem to appear as regularly when classifying acne or large pores.

Further analysis of Table 2 shows a clear improvement of the modified Multi‐scale Azimuthal Projection Distance Image over the original formulation. The M‐APDI with depth 1, where the sole difference from the original formulation is the introduction of a new way of computing the pixels as a function of the two projection coordinates, introduces improvement in the classification results. These improvements become even more significant as the M‐APDI pyramid goes deeper.

The Local Orientation Patterns, even though not multi‐scale, produce comparable results to the M‐APDI. Furthermore, comparing the results yielded by the first and second proposed pattern function show clear improvement using the second pattern function over the first on classifying wrinkle and pore visibility while the first pattern function does slightly better on classifying acne.

The BTF Texton and our proposed Rotation Fields methods yield the highest performance rates. The BTF Texton gives somewhat better classification of wrinkles and acne than the Rotation Fields, with average improvements of 0.050 on wrinkle and 0.046 on acne. However, the Rotation Fields yield slightly better results on classifying pore visibility with an average improvement of 0.033. This can be explained by the high and low frequency separation performed in the Rotation Fields and not in the BTF Texton. Furthermore, the data needed to compute the Rotation Field (ie normal map) have a more compact representation. Even though it is trivial to recover surface normals from BTF data or generate BTF data from surface normals, it is more practical to store or distribute a data set in the form of normal maps as BTF databases are known to be demanding in storage capacity.

## 6 CONCLUSIONS

In this paper, we have explored three new methods of characterising the 3D nature of surface texture and have applied these to facial skin texture analysis. In contrast to image‐based methods, which use BTF data, the surface texture descriptors proposed in this paper operate directly on the captured surface microgeometry in the form of dense surface normals. The performances of these are evaluated on classifying common skin conditions (wrinkles, large pores, acne) and compared against state‐of‐the‐art methods represented by a BTF Texton‐based approach. We have also compared the performances of traditional two‐dimensional texture measures (LBPs and Gabor filter banks) and simplistic extensions of these to the 3D space.

The experiments show that, of the three proposed methods, Rotation Fields produce the best classification results with average F‐measures of 0.86, 0.91 and 0.86 classifying, respectively, wrinkles, pores and acne. The BTF Texton‐based method performs better than the Rotation Fields on classifying wrinkles and acne with, respectively, F‐measures of 0.91 and 0.90. However, on classifying pores the Rotation Fields give somewhat better results with 0.91 against 0.87. This suggests that the Rotation Fields are more efficient at characterising conditions associated with high frequency visual presentation, such as pores. Conditions associated with coarser, lower frequency visual features such as wrinkles and acne are better classified using the BTF Texton. This can be explained by the high and low frequency separation performed in the Rotation Fields method and not in the BTF Texton method. Other benefits of the Rotation fields should be considered: these include a more compact representation of both feature vectors and data sets and the ability to take advantage of recent advances made in 3D surface capture techniques.

The good classification results yielded by the multi‐layer perceptron hints at potential extra gain if, instead of hand crafting the 3D surface texture descriptors, techniques such as a Convolutional Neural Network were used to learn these. It is indisputable that the back propagation involved in such a network considerably benefits the features learnt in the convolutional layers. But training a Convolutional Neural Network requires a much more extensive data set than our limited set of facial region captures, hence, the relevance of hand crafting our convolutional layer and passing the results on to a multi‐layer perceptron. However, extending our data set and trying to learn a set of meaningful convolutional nodes for 3D surface texture analysis and synthesis remains a very good candidate for future work.
